# Cardiac Computed Tomography as a Method of Diagnosing the Type of Cardiac Tumor—Example of Interatrial Septal Lipoma Filling the Right Atrium

**DOI:** 10.3390/diagnostics14222496

**Published:** 2024-11-08

**Authors:** Paweł Gać, Arkadiusz Jaworski, Agnieszka Parfianowicz, Alicja Surma, Anna Jakubowska-Martyniuk, Aleksandra Żórawik, Rafał Poręba

**Affiliations:** 1 Centre for Diagnostic Imaging, 4th Military Hospital, 50-981 Wroclaw, Poland; 2 Department of Environmental Health, Occupational Medicine and Epidemiology, Wroclaw Medical University, 50-367 Wroclaw, Poland

**Keywords:** cardiac computed tomography angiography, cardiac tumor, lipoma, right atrium

## Abstract

Cardiac tumors present substantial diagnostic challenges due to their diverse manifestations and similarity to other cardiac pathologies. Cardiac lipomas are rare tumors that originate from adipose cells and can develop in any location within the heart. Cardiac lipomas account for 2.4% of all primary cardiac tumors. Most lipomas are located within the cardiac chambers. Among the lipomas occurring within the cardiac chambers, the most common localization is the right atrium. Currently, the gold standard for imaging cardiac tumors is cardiac magnetic resonance (CMR). Despite the significant advantages of CMR, cardiac computed tomography angiography (CCTA) continues to be a valuable technique when CMR is either unavailable or contraindicated. In some cardiac tumors, CCTA can identify the type of tumor. A classic example of this type is a lipoma. We present images of a large interatrial septal lipoma filling the right atrium diagnosed by CCTA in a 57-year-old female Caucasian patient. In summary, CCTA effectively identifies lipomas’ characteristic features and provides crucial information for appropriate management.

**Figure 1:**
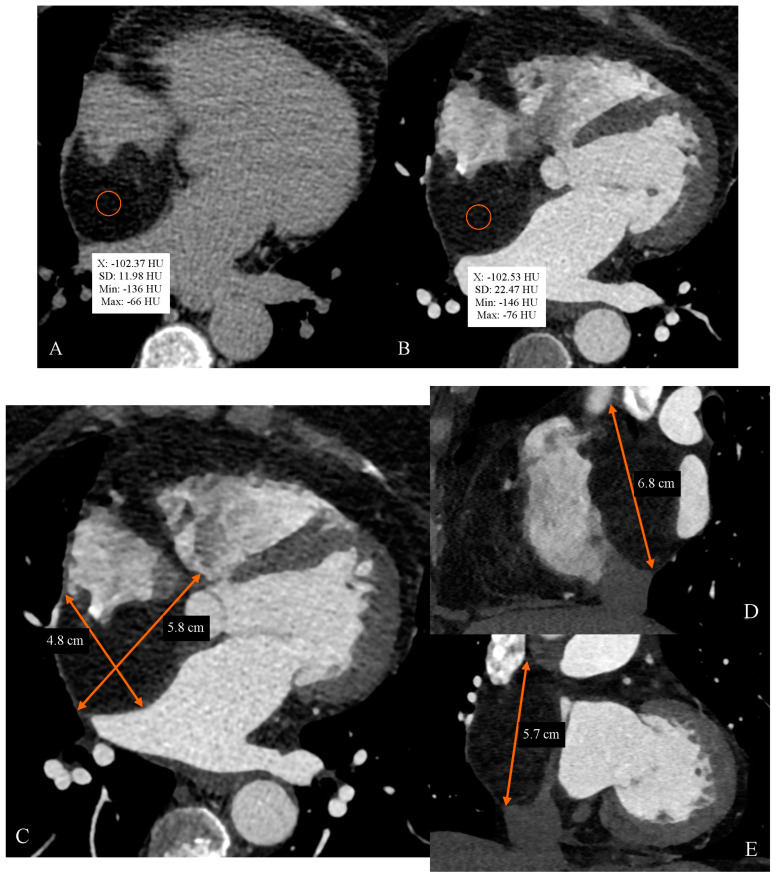

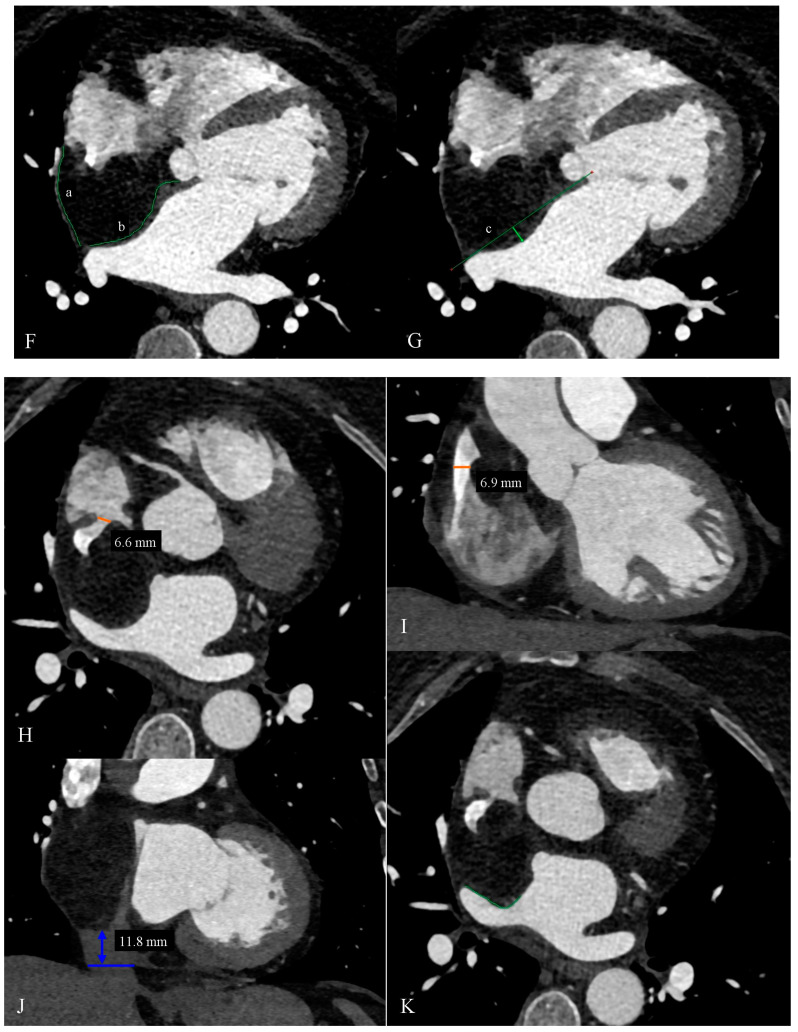

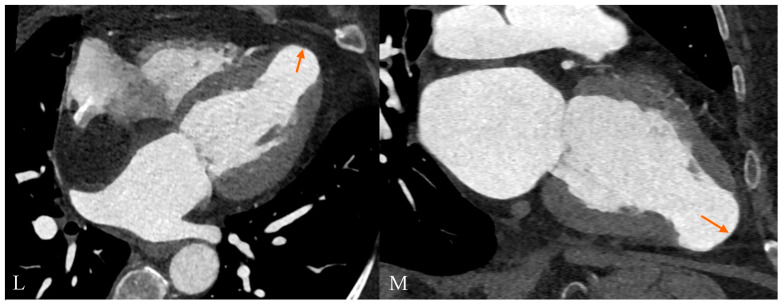
A 57-year-old Caucasian woman with a history of conservatively treated myocardial infarction 20 years ago, arterial hypertension, type 2 diabetes, hypercholesterolemia, obesity, bronchial asthma, goiter, and smoking presented to the hospital emergency department with chest pain. She reported palpitations, increased fatigue, decreased exercise tolerance, and edema. Troponin I was negative. The patient was admitted to the cardiology department, and invasive coronary angiography excluded the presence of new stenoses in the coronary arteries requiring revascularization. A screening echocardiography revealed the presence of an additional structure in the right atrium, and a decision was made to perform cardiac computed tomography angiography (CCTA). (**A**) CCTA native phase in 3.0-mm-thick slices, axial view. (**B**) CCTA angiographic phase after intravenous administration of 65 mL of iodinated nonionic contrast agent in 0.6-mm-thick slices, axial view. In the enlarged right atrium, there was a large, polycyclic, homogeneous tumor with a density of approximately −100 HU without post-contrast enhancement. (**C**) CCTA angiographic phase, axial view. (**D**) CCTA angiographic phase, sagittal view. (**E**) CCTA angiographic phase, coronal view. The tumor mass significantly reduces the lumen of the right atrium. The tumor size is approximately 5.8 × 4.8 cm in axial view and approximately 6.8 cm in craniocaudal dimension. (**F**) CCTA angiographic phase, axial view. The tumor mass is partially adherent to the free wall of the right atrium in its posterolateral part (green curved line marked with the letter “a”). The tumor mass is adherent to the atrial septum along its entire length (green curved line marked with the letter “b”). (**G**) CCTA angiographic phase, axial view. The tumor mass displaces the atrial septum toward the left atrium (the green straight line marked with the letter “c” indicates the plane of the atrial septum; the green arrow indicates the displacement of the atrial septum). (**H**) CCTA angiographic phase, axial view. (**I**) CCTA angiographic phase, coronal view. The tumor mass reduces the size of the outlet of the superior vena cava to the right atrium to minimal dimensions of approximately 0.7 × 0.7 cm (dimensions marked with orange lines). (**J**) CCTA angiographic phase, coronal view. The tumor mass penetrates toward the outlet of the inferior vena cava into the right atrium (minimum distance of about 1.2 cm). The blue line indicates the plane of the outlet of the inferior vena cava into the right atrium. The blue arrow indicates the minimum distance of the tumor mass from the outlet of the inferior vena cava to the right atrium. (**K**) CCTA angiographic phase, axial view. The bulging wall of the right atrium displaces the distal segment of the right upper pulmonary vein (green curved line). The morphology of the tumor in the CCTA is highly suggestive of a neoplastic lesion of the lipoma type. (**L**) CCTA angiographic phase, cardiac views, four-chamber view. (**M**) CCTA angiographic phase, cardiac views, two-chamber view. Co-occurrence of post-infarction aneurysmal thinning of the left ventricular myocardium in the apical septal segment, the inferior apical segment, and the apex (marked by orange arrows). The patient had a history of myocardial infarction due to occlusion of the distal left descending artery (LAD), which was treated conservatively. The dimensions of the aneurysm are stable in long-term follow-up; the aneurysm remains stable under further investigation. The patient qualified for planned cardiac surgery for the resection of the interatrial septal tumor filling the right atrium. During cardiac surgery, the tumor was removed. The atrial septum and part of the right atrial free wall were reconstructed using a Gore-Tex patch. Histopathological examination confirmed the type of tumor. Cardiac tumors present substantial diagnostic challenges due to their diverse manifestations and similarity to other cardiac pathologies. They can be divided into primary tumors and secondary tumors. The former are extremely rare, with an approximate incidence of 0.02% [1] or 1:2000 autopsies [2]. Approximately 90% of primary cardiac tumors are benign [2,3]. The latter arise as a result of metastases and are more common, with a prevalence of 1:100 autopsies [2,4]. Timely and precise diagnosis is essential for optimal treatment and management, as it facilitates early intervention and appropriate therapeutic decisions. The primary methods for diagnosing cardiac tumors include cardiac magnetic resonance (CMR), cardiac computed tomography angiography (CCTA), transthoracic echocardiography (TTE), transesophageal echocardiography (TEE), and contrast-enhanced ultrasonography (CEUS). These techniques provide detailed visualization and characterization of cardiac masses. They allow for the assessment of tumor size, location, morphology, and tissue characteristics, thereby guiding clinical decisions effectively [4,5,6]. Notably, a multimodality imaging approach in the characterization of cardiac tumors offers the most complex and complementary insights [7,8,9,10]. TTE is a widely available, noninvasive technique, and therefore, it serves as the initial imaging modality. The sensitivity and specificity of this technique can reach 90% and 95%, respectively [11]. Echocardiography images assess the tumor size, location, contours, site of attachment, and mobility and provide information about obstruction to the circulation [12]. Furthermore, TTE may serve as the final imaging method in some situations, such as in certain cases of myxoma [13]. On the other hand, TEE is more informative, as it provides additional imaging planes and higher resolution [14]. However, the insertion of the probe through the esophagus is invasive. CEUS visualizes contrast perfusion and thus reveals tumor vascularity [4,15]. This feature enables the differentiation of neoplasms from thrombi [6,16]. Nevertheless, echocardiographic modalities have restricted fields of view and are highly dependent on the experience and skill of the operator [4]. Currently, the gold standard for imaging cardiac tumors is cardiac magnetic resonance (CMR). CMR is the best available noninvasive diagnostic tool for evaluating heart tumors [17,18,19]. It requires the application of gadolinium and does not expose the patient to radiation. Compared with other imaging techniques, CMR has the highest soft tissue contrast resolution [20]. CMR provides information about topographic relations and the extent of the tumor’s spread to adjacent structures [2]. Additionally, it allows for tissue characterization. T1-weighted sequences are effective in detecting tumors with a high content of fatty tissue, such as lipomas or lipomatous hypertrophy. Conversely, T2-weighted sequences are helpful in diagnosing masses containing a large amount of water, such as myxomas or pericardial cysts [6,17]. After the administration of gadolinium, CMR shows specific enhancement patterns—absent, early, or delayed—that are helpful in identifying the type of tumor. First-pass gadolinium perfusion sequences suggest the presence of a highly vascularized tumor. Delayed gadolinium uptake can be categorized into homogeneous uptake, as seen in fibromas, or heterogeneous uptake, as seen in angiosarcomas [6,18,19]. Similar to CMR, CCTA enables detailed visualization with high spatial resolution of tumor morphology and its relationship to adjacent structures, which is essential for effective diagnostic and therapeutic planning [4,21]. Consequently, it is essential in detecting secondary cardiac tumors with primary malignancy external to the heart. Contrast enhancement supports the identification of highly vascularized tumors. The procedure can be ECG-gated, which controls the motion artifact and provides additional assessment of cardiac function. Compared with CMR, CCTA delivers an evaluation of calcifications and is less expensive. Furthermore, it is essential in detecting lipomas, as fat is sufficiently observed on CT [5]. Lipomas present a low-attenuation signal [6,22]. Despite the reduction in the radiation dose with technological advancements [23], patients remain exposed to ionizing radiation, which remains a significant drawback. Moreover, as opposed to CMR, the temporal and soft-tissue resolution remains low [4]. Despite the significant advantages of CMR, CCTA continues to be a valuable technique when CMR is either unavailable or contraindicated [7,21]. In some cardiac tumors, CCTA can identify the type of tumor. A classic example of this type is a lipoma. In the case of homogeneous, low-density (<−50 HU), and well-defined (encapsulated) tumors, radiological diagnosis of lipoma using computed tomography is characterized by sensitivity and specificity like magnetic resonance [24]. Cardiac lipomas are rare, benign tumors that originate from adipose cells and can develop in any location within the heart [25]. The first case of cardiac lipoma was reported in 1856 by Albers when cardiac lipomas were often discovered incidentally during autopsies or cardiothoracic surgeries [26]. This was largely because, in most cases, the tumor did not present with any clinical symptoms [27]. The specific number of reported cases can vary widely depending on the source, but given their rarity, there are likely only a few hundred documented cases in the medical literature worldwide. According to a report from the Japanese Circulation Society, cardiac lipomas account for 2.4% of all primary cardiac tumors [28]. The tumor is usually composed of white adipose cells or, less commonly, of fetal brown fat, which are typically encapsulated by connective tissue. The size and weight of cardiac lipomas vary, with reported measurements including 5 × 5 cm and a weight of 50 g, 14 × 11 cm and a weight of 450 g, and even a giant tumor measuring 18 × 16 × 19 cm and weighing 3.75 kg [29,30,31]. Most tumors (53.1%) are located within the cardiac chambers; other locations include the pericardium (32.5%), myocardium (10.7%), and other structures (3.7%). Among the tumors occurring within the heart chambers, the most common localization is a lipoma of the right atrium (RA) (33.3%) [32,33]. The etiology of cardiac lipoma remains incompletely understood. Genetic alterations predominantly affecting the HMGA2 gene are frequently observed in extracardiac lipomas [33]. By contrast, such cytogenetic abnormalities are rarely detected in cardiac lipomas [34]. Cardiac lipomas can develop at any stage of life, from the fetal period to individuals in their 80s. However, most cases are reported in the range of 40–70 years of age, with no difference in distribution between genders [32]. Although the tumors are considered benign and usually asymptomatic, they may present with symptoms due to their infiltration of adjacent structures. Clinical manifestations may vary from obstruction, arrhythmias such as atrial fibrillation (AF), atrial flutter, syncope, and hemodynamic instability to SCD [24,34]. Pericardial lipomas are usually asymptomatic unless they grow large enough to compress cardiac structures. By contrast, intracardiac lipomas can cause significant symptoms by obstructing blood flow or interfering with heart valve function, potentially leading to arrhythmias or heart failure. RA lipoma may present with symptoms such as dyspnea (35%), including dyspnea on exertion, followed by chest pain (15%) and palpitation (12%) [10,29,32,35,36]. The mechanisms responsible for these symptoms include the direct blockage of blood flow within the heart, the impairment of cardiac valve function, the obstruction of the superior and inferior vena cava, and the involvement of the phrenic nerve [37]. A rarer variant of cardiac lipoma is the invasive lipoma, which has a more pronounced ability to infiltrate adjacent structures, including the myocardium, and cause obstruction, arrhythmias, and hemodynamic instability. As a result, it is associated with higher mortality [38]. Nowadays, thanks to advances in imaging studies, many asymptomatic cardiac tumors are detected during routine examinations [39]. Echocardiography plays an important initial role in the screening and evaluation of cardiac lipoma [35]. However, CT and CMR are indispensable for accurate diagnosis and comprehensive evaluation [40]. Combining different imaging modalities enables clinicians to make the best decision for the patient [10,24]. The differential diagnosis of cardiac lipoma includes other primary benign cardiac tumors, liposarcoma, thrombus, and lipomatous hypertrophy of interatrial septum (LHIS). Other primary cardiac tumors or thrombi are characterized by a different tissue morphology in imaging studies; for example, in computed tomography, they have a higher and often significantly more heterogeneous density than lipoma. Liposarcoma, in addition to the fat component, contains a soft tissue component, which in computed tomography is characterized by a density of several tens of HU. In addition, liposarcoma usually presents with the invasion of neighboring anatomical structures [41]. LHIS, which is not a true neoplasm, is characterized by the absence of a capsule, brown fat content, sparing of the fossa ovalis (dumbbell appearance of the atrial septum), and fluorodeoxyglucose (FDG) uptake on positron emission tomography (PET) [42]. In the case of differentiation between cardiac lipoma and LHIS, histopathological examination is crucial. Lipomas are encapsulated and contain neoplastic mature adipocytes but do not contain brown fetal fat cells. LHIS is characterized by the infiltration of mature adult-type or fetal fat cells between myocardial fibers with the absence of capsule [43]. LHIS may be asymptomatic, but it may also be associated with arrhythmia. Cardiac lipomas are generally not associated with arrhythmia, although cases of arrhythmia have also been described in the case of large lipomas located near the cardiac conduction system [43]. The method of choice in treating symptomatic cardiac lipoma is surgical excision, while asymptomatic tumors are more often managed conservatively [31,38,39]. The decision should be highly individualized, with consideration of the risk–benefit ratio and shared decision-making [35]. In conclusion, CCTA is a valuable imaging modality for aiding the diagnosis of cardiac tumors. CCTA provides detailed visualization of tumor morphology and its relationship with adjacent cardiac structures, essential for accurate diagnosis and therapeutic planning. Lipomas filling the RA, typically benign, can cause significant symptoms based on size and location. CCTA effectively identifies lipomas’ characteristic features and provides crucial information for appropriate management, whether surgical or conservative.

## Data Availability

The data presented in this article are available upon request from the corresponding author.
